# Survival status and predictors of mortality from severe community-acquired pneumonia among under-five children admitted at Debre Tabor comprehensive specialized hospital: a prospective cohort study

**DOI:** 10.3389/fped.2023.1141366

**Published:** 2023-06-05

**Authors:** Amare Kassaw, Gashaw Kerebih, Shegaw Zeleke, Ermias Sisay Chanie, Nigatu Dessalegn, Berihun Bante, Asefa Ageghehu Teshome, Bogale Chekole, Belete Gelaw, Wubet Alebachew Bayih, Aragaw Tesfaw, Dejen Getaneh Feleke, Demewoz Kefale, Molla Azmeraw, Aynadis Chanie, Getaneh Awoke, Natnael Moges

**Affiliations:** ^1^ Department of Pediatrics and Child Health Nursing, College of Health Sciences, Debre Tabor University, Debre Tabor, Ethiopia; ^2^ Department of Adult Health Nursing, College of Health Sciences, Debre Tabor University, Debre Tabor, Ethiopia; ^3^ Department of Pediatrics and Child Health Nursing, College of Medicine and Health Sciences, Mizan Tape University, Mizan Tape, Ethiopia; ^4^ Department of Biomedical Science, College of Health Sciences, Debre Tabor University, Debre Tabor, Ethiopia; ^5^ Department of Pediatrics and Child Health Nursing, College of Medicine and Health Sciences, Wolkite University, Wolkite, Ethiopia; ^6^ Department of Pediatrics and Child Health Nursing, College of Medicine and Health Sciences, Wolayta Sodo University, Wolayta, Ethiopia; ^7^ Department of Maternal and Neonatal Health Nursing, College of Health Sciences, Debre Tabor University, Debre Tabor, Ethiopia; ^8^ Department of Epidemiology and Biostatics, College of Health Sciences, Debre Tabor University, Debre Tabor, Ethiopia; ^9^ Department of Pediatrics and Child Health Nursing, College of Health Sciences, Woldia University, Woldia, Ethiopia; ^10^ Department of Comprehensive Nursing, Bahir Dar University, Tibebe Gion Specialized Hospital, Bahir Dar, Ethiopia; ^11^ Department of Epidemiology, Debre Tabor Health Sciences College, Debre Tabor, Ethiopia

**Keywords:** under-five children, survival status, predictors, pneumonia, Ethiopia

## Abstract

**Background:**

Globally, Pneumonia continues to be the leading cause of mortality among under-five children. Ethiopia ranks fourth out of 15 countries worldwide in terms of the highest death rate of under-five children due to severe community-acquired pneumonia (SCAP). However, to date, there is no recent study that shows survival status and predictors of mortality from SCAP. Therefore, this study aimed to determine survival status and predictors of mortality from this dangerous disease among under-five children.

**Methods:**

A facility-based prospective cohort study was conducted from 1 November 2021 to 31 October 2022 at Debre Tabor comprehensive specialized hospital. All under-five children with SCAP admitted during the study period were included. Participants were selected using a systematic sampling technique. The collected data were coded, edited, and entered into epi-data version 4.2 and then exported to STATA version 17 for further analysis. The Kaplan Meier failure estimate with log-rank test was employed to determine the survival estimates. A cox-proportional hazard regression model was fitted to identify significant variables.

**Results:**

The overall incidence density rate of mortality was 5.7 /1000 children with a median hospital stay of 8.2 days. Heart disease (AHR: 4.37; 95%CI: 1.68–11.32), previous admission of SCAP (AHR: 3.87; 95% CI: 1.31–11.43), WFL < −3Z score (AHR: 3.57; 95% CI: 1.02–12.42), impaired consciousness level at admission 3.41(1.14–10.19), and pleural effusion (AHR: 3.42; 95%CI: 1.18–9.93) were significant predictors of mortality.

**Conclusion:**

In this study, the survival probability of children with SCAP was low. Children with heart disease, previous admission of SCAP, WFL < −3Z score, impaired consciousness level at admission, and pleural effusion had low survival. Therefore, much emphasis is needed on children with SCAP, particularly those with identified predictors.

## Introduction

Pneumonia is defined as an inflammation of the parenchymal structure of the lungs, and it is the most common infectious cause of death globally among under-five children (1). It is broadly classified as community-acquired and hospital-acquired pneumonia based on the setting in which infection occurs (2). Community-acquired pneumonia (CAP) refers to an infection of the acute lower respiratory tract that occurs outside of a health facility or one that develops within 48 hours after admission to a health facility (3). It is also classified as “pneumonia” and “severe pneumonia” based on clinical features. Patients with fast breathing or chest indrawing are classified as having “pneumonia”, whereas pneumonia with any general ganger sign is categorized as “severe pneumonia (4). The etiology of community-acquired pneumonia is bacteria, viruses, fungi, and parasites. CAP usually causes children to become ill with high-grade fever and fast breathing (5, 6).

Despite the implementation of safe, effective, and affordable interventions, pneumonia continues to be the leading cause of mortality among under-five children globally (4). It is estimated to be responsible for approximately 16% of the 5.6 million under-five deaths, killing around 900,000 children (7) and accounting for approximately 1 in 5 child deaths worldwide (2). The majority of these deaths are due to severe community-acquired pneumonia (SCAP) (8). Even though mortality from childhood SCAP has decreased in the last few decades in developed countries, the disease has continued to be a common cause of morbidity and mortality (2). Evidence has estimated that in 2015, about 80% of these deaths occurred in South Asia and sub-Saharan Africa and almost half occurred in just five countries: India, Nigeria, Pakistan, Democratic Republic of Congo, and Ethiopia (9, 10). In 2016 nearly half a million children under the age of five died of pneumonia in sub-Saharan Africa including Ethiopia (11).

Although Ethiopia has implemented different strategies to halt child morbidity and mortality due to SCAP, recent evidence has shown that it ranks fourth out of 15 countries in terms of the highest death rate of under-fives worldwide (12).

Studies showed that wasting, large family size, non-exclusive breast feeding, younger maternal age (6), low birth weight, air pollution, parental smoking, being unvaccinated, overcrowding, lack of a separate kitchen, and lack of maternal education were a risk factors of pneumonia (13–16). However, these studies did not indicate the survival status using time to event data analysis and whether those identified risk factors predict mortality. This study was expected to fill the gap using a prospective cohort study design to identify predictors of mortality in under-five children with SCAP to assist clinicians in prioritizing interventions.

## Methods

### Study area

This study was conducted at Debre Tabor Comprehensive Specialized Hospital (DTCSH). DTCSH is found in Debre Tabor town which is the administrative town of the South Gondar Zone of Amhara regional state. It is located approximately 565 km Northwest of Addis Ababa, the capital city of Ethiopia, and 100 km from Bahir dar, the capital city of Amhara regional state. The governmental health institutions in the town include one Specialized Comprehensive Hospital and three health centers. The hospital's pediatrics department has pediatric wards with 45 beds and an emergency unit with 8 resuscitation beds. It has a total of 35 health professionals (6 pediatricians, 4 general practitioners and 25 nurses). The average monthly and annually case flow of under-five children with community acquired pneumonia was 97 and 1,164 admitted to the pediatrics ward respectively.

### Study design and participant characteristics

A facility-based prospective cohort study design was conducted. All under-five children diagnosed with severe community acquired pneumonia who were admitted during the 1 November 2021 to 31 October 2022 period at DTCSH were included in the study.

### Sample size determination and sampling procedure

The sample size was calculated using Epi-info Version7 statistical software by considering factors like antibiotics received at first contact, Oxygen saturation on admission, past history of ARI/diarrhea in last 3 months, and exclusive breast feeding till 6 months (17). Antibiotics received at first contact was considered as an independent predictor since it produces a maximum sample size. The total annually average admission of under-five children diagnosed with SCAP at DTCSH as per the 2020/2021 report was 1,164, which was considered to be the study populations. All the eligible under-five children with severe community acquired pneumonia during the study period were included. The participants were selected using a systematic random sampling technique using K intervals (N/n = 1,164/590 = 1.97∼2).

### Operational definition of terms

**Censored:** Under-five children diagnosed with SCAP at the pediatric ward with predictors but who recovered and were discharged home, discharged against medical advice, lost to follow up, or transferred out to other health institutions without knowing the outcome.

**Survival status:** The final outcome of under-five children with severe community-acquired pneumonia (either death or censored).

**Event:** Death.

**Survival time:** Measure of the follow-up time from a defined starting point/from admission of under-five children diagnosed with SCAP to pediatrics ward up to the occurrence of the event**.**

**Danger signs:** Loss of consciousness, abnormal body movement, vomiting, convulsion, and inability to feed in addition to SCAP (18).

**Fully vaccinated:** Full vaccination includes all children who had obtained BCG (bacillus calmette–guérinvaccine) and OPV0 (oral polio vaccine) at birth, pentavalent1 [DPT-hepB-Hib (diphtheria, Pertussis, tetanus, hepatitis B and Haemophilus influenza type b)], OPV1, PCV1, and Rota1 at 6 weeks; pentavalent 2, OPV2, Rota2, and PCV2 at 10 weeks; pentavalent 3, PCV3, and IPV at 14 weeks; and measles vaccine at 9 and 15 months (11).

**Co-morbidity:** Any disease condition (acute or chronic) present at admission in addition to SCAP, which includes hyperactive airway disease (childhood asthma), retroviral infection, Tuberculosis, acute gastroenteritis, Pertussis, anemia, meningitis, measles, bronchitis, heart disease, and urinary tract infection (19). A detailed description is listed in (supplementary file).

### Data collection tools and procedure

The data were collected by three trained professional nurses using a structured interviewer administered questionnaire for interviewing mothers/guardians and checklists. The questionnaire has been adapted and modified from studies in Ethiopia, West Bengal, and India (11, 17, 19–21). Furthermore, structured checklists were adapted from the aforementioned studies to extract data on some parental and child related factors to the death of children with SCAP. The checklists and interviewer-administered questionnaires included socio-demographic, co-morbidities, feeding, and treatment related factors. The starting point for prospective follow-up was the time from first admission date and the endpoint was the date of death and censored.

The study participants were selected based on the eligibility criteria. The survival status of patients was obtained from the medical records. Survival time was calculated as the time between the date of admission to the date of death, censored, or the end of the study.

### Data quality assurance

The questionnaires were first prepared in English and then translated into the local language, Amharic, for suitable data collection. One-day training and clear orientation were first provided for the data collectors and supervisors on the subjects of pretesting and the process of data collection. Before the actual data collection, pretests were done using 30 eligible mother-baby dyads (5% of sample size) at Mekane Eyesus primary hospital to evaluate the clarity of questions, validity of the instrument and reaction of the respondents to the questions. During data collection, data collectors were closely monitored and guided by two MSc pediatric nurse supervisors. The collected data were reported to the principal investigator regularly.

### Data processing and analysis

Before analysis, data were cleaned, edited, and coded. Any error identified at this time was corrected after review of the original data using the coded numbers. Data was entered into Epi-Data Version 4.2, and then exported to STATA 17 for further analysis. The continuous data, depending on the distribution, were described either in mean and standard deviation or median. Frequency distribution was used for categorical data.

Finally, the outcome of each participant was dichotomized into censored or death. The incidence density rate (IDR) was calculated for the entire study period. Subsequently, the number of mortality within the follow-up period were divided by the total person-time at risk on follow up and reported. Kaplan Meir was used to estimate mean survival time and cumulative survival probability and log-rank tests was used to compare the survival time between different categories of explanatory variables.

Before running Cox proportional hazard regression model, multi-collinearity was checked using variance inflation factor (VIF) with pair-wise correlation, and those variables with a strong correlation were removed from the analysis. Residuals were also checked using goodness-of-fit tests by Cox Snell residuals, which satisfy the model test.

Proportional Hazard assumption was checked using the Schoenfeld residual statistical test.

A bi-variable Cox-proportional hazard regression model was fitted for each explanatory variable. Variables with *p*-values < 0.25 in the bi-variable analysis were fitted to the multivariable Cox-proportional hazards regression model. Hazard ratios with 95% confidence intervals and *p*-values ≤0.05 will be used to measure the strength of association and to identify statistically significant predictors.

### Ethical consideration

Ethical clearance was obtained from Debre Tabor University, College of Health Sciences, and the Ethical Clearance Review Committee with protocol number Dtu/RP/186/15. Then, an official letter was submitted to DTCSH. After explaining the study, voluntary written informed consent was obtained from each mother. Since the study was conducted through a face-to-face interview and chart review, the individual participants were not exposed to any harm. Moreover, the mothers were told that the information they gave would be treated with complete confidentiality and does not cause any harm. Data collectors applied infection prevention techniques like proper hand washing, wearing gloves, and alcohol rubbing of instruments before touching children.

## Results

### Socio-demographic factors

A total of 580 study participants were followed in this study, resulting in response rates of 98.3%. More than half of the participants, a total of 373 (64.3%), came from a rural area. The majority of the study participants, a total of 377 (65.0%), were aged less than 24 months. Nearly half of the mothers 285 (49.1%) were in the age range of 25–34, and 169 (29.1%) had primary educational status (Table 1).

**Table 1:** Socio-demographic factors of children with SCAP admitted at DTCSH, North central Ethiopia, 2022.

Variables	Categories	Frequency *n* (%)
Age of child in month	<24	377 (65.0)
	≥24	203 (35.0)
Sex of the child	Male	292 (50.3)
	Female	288 (49.7)
Age of the mothers	15–24	63 (10.9)
	25–34	285 (49.1)
	≥35	232 (40.0)
Occupation of the mothers	Unable to read and write	157 (27.1)
	Primary (1–4)	105 (18.1)
	Primary (5–8)	169 (29.1)
	Secondary (9–12)	99 (17.1)
	College and above	50 (8.6)
Occupation of mothers	Housewife	205 (35.3)
	Farmer	144 (24.8)
	Government employee	100 (17.2)
	Merchant	74 (12.8)
	Private gainful work	35 (6.1)
	Others	22 (3.8)
Number of family members	<3	144 (19.7)
	3–5	232 (40.0)
	>5	234 (40.3)
Vaccination status	Fully vaccinated	282 (48.6)
	Partially vaccinated	104 (17.9)
	Up to date	165 (28.5)
	Unvaccinated	29 (5.0)
Residence	Urban	207 (35.7)
	Rural	373 (64.3)

### Clinical conditions and co-morbid factors

The common co-morbidities identified during admission of children were heart disease (25.2%), hypoglycemia (20%), diarrhea (25%) pulmonary TB (8.8%), and altered consciousness level at admission (12.5%) (Table 2).

**Table 2:** Clinical conditions and major co-morbidities of children with SCAP admitted at DTCSH, North central Ethiopia, 2022 (*n* = 580).

Variables	Categories	Frequency *n* (%)
HIV/AIDS	Reactive	30 (5.2)
	Non-reactive	381 (65.7)
	Unknown	169 (29.1)
Pulmonary TB	Yes	51 (8.8)
	No	529 (91.2)
Heart disease	Yes	146 (25.2)
	No	434 (74.8)
Measles	Yes	76 (13.1)
Previous admission of SCAP	No	504 (86.9)
	Yes	145 (25.0)
	No	435 (75.0)
Treatment failure	Yes	169 (29.1)
	No	411 (70.9)
Hypoglycemia	Yes	116 (20.0)
	No	464 (80.0)
Shock	Yes	66 (11.4)
	No	514 (88.6)
Chest in drawing	Yes	228 (39.3)
	No	352 (60.7)
Oxygen saturation	<90%	416 (71.7)
	≥90%	164 (28.3)
Diarrhea	Yes	145 (25.0)
	No	435 (75.0)
Anemia	Yes	100 (17.2)
	No	480 (82.8)
Fast breathing	Yes	496 (85.5)
Admission pulse rate	No	84 (14.5)
	Altered	338 (58.3)
	Normal	242 (41.)
Admission respiratory rate	Altered	466 (80.3)
Temperature at admission	Normal	114 (19.7)
Developmental delay	Altered	311 (53.6)
Status at admission	Normal	269 (46.4)
	Yes	99 (17.1)
	No	481 (82.9)
	Altered	94 (16.2)
	Conscious	486 (83.8)

### Nutritional status and laboratory finding related factors

More than half of the patients 310 (53.5%) had abnormal WBC count and 295 (49.1%) of them also had WHZ-score < −3. Whereas, most of the children 406 (70.0%) with SCAP had HAZ-score ≥ −3 (Table 3).

**Table 3:** Nutritional status and laboratory finding related factors of children with SCAP admitted at DTCSH, North central Ethiopia, 2022.

Variables	Categories	Frequency *n* (%)
WBC count	Normal	128 (22.0)
	Abnormal	310 (53.5)
	Not done	142 (24.5)
RBC count	Normal	195 (33.6)
	Abnormal	259 (44.7)
	Not done	126 (21.7)
x-ray	Normal	74 (13.0)
	Abnormal	133 (23.4)
	Not done	362 (63.6)
WHZ	Z-score < −3	295 (49.1)
	Z-score ≥ 3	285 (50.9)
HAZ	Z-score < −3	174 (30.0)
	Z-score ≥ −3	406 (70.0)

### Feeding and treatment related factors

Most of the children, a total of 533 (91.9%), had taken antibiotics. More than half of the children, a total of 333 (57.4%), had exclusively been breast fed until 6 months (Table 4).

**Table 4:** Feeding and treatment related factors of children with SCAP admitted at DTCSH, North central Ethiopia, 2022.

Variables	Categories	Frequency *n* (%)
EBF until 6 month	Yes	333 (57.4)
	No	247 (42.6)
Starting of complementary feeding	Before 6 month	213 (39.9)
	At 6 month	278 (52.1)
	After 6month	43 (8.0)
Taking of antibiotics	Yes	533 (91.9)
	No	43 (8.1)
Vitamin A given	Yes	188 (32.4)
	No	392 (67.6)
Vitamin D given	Yes	225 (38.8)
	No	355 (61.2)
Iv fluid given	Yes	495 (85.3)
	No	85 (14.7)

### Overall survival estimate of children with severe community acquired pneumonia

**Figure 1:**
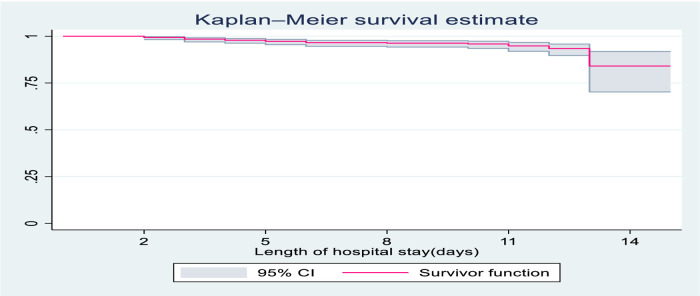
Overall Kaplan-Meier survival estimate of children with SCAP admitted to DTCSH, from 2021 to 2022, North central Ethiopia, 2022.

The overall follow up of study participants were 4,716 child-days with an incidence rate of 5.7 deaths per 1,000 child-days (95% CI: 3.9, 8.3), and the median duration of hospital stay was 8.2 days. The survival probability of children with severe community-acquired pneumonia was estimated using Kaplan-Meier estimates. The survival probability at the 3rd day of admission was high at almost 99.3% with a standard error of 0.0035 (95% CI: 98.2, 99.7). On the 7th day of hospital stay, the survival probability of SCAP children was also found to be 96.4% with a standard error of 0.0083 (95% CI: 94.4, 97.8). At the end of the 15th day the overall survival probability was 79% with a standard error of 0.0726 (95% CI: 60.3 89.6) (Figure 1).

Regarding the Incidence Rate (IR) of time to death, children with heart disease had a lower survival time with an incidence rate of death (IR 20.8/1,000) child-days compared to those with no heart problems [incidence rate of (IR 2.4/1,000) child-days]. The study also showed that children whose WFL < −3Z score had reduced survival time with incidence rate of death 10.7/1,000 compared to whose WFL ≥ −3Z score with incidence rate of 4.8/1,000 child-days.

#### Survival function and comparison of survivorship functions

**Figure 2:**
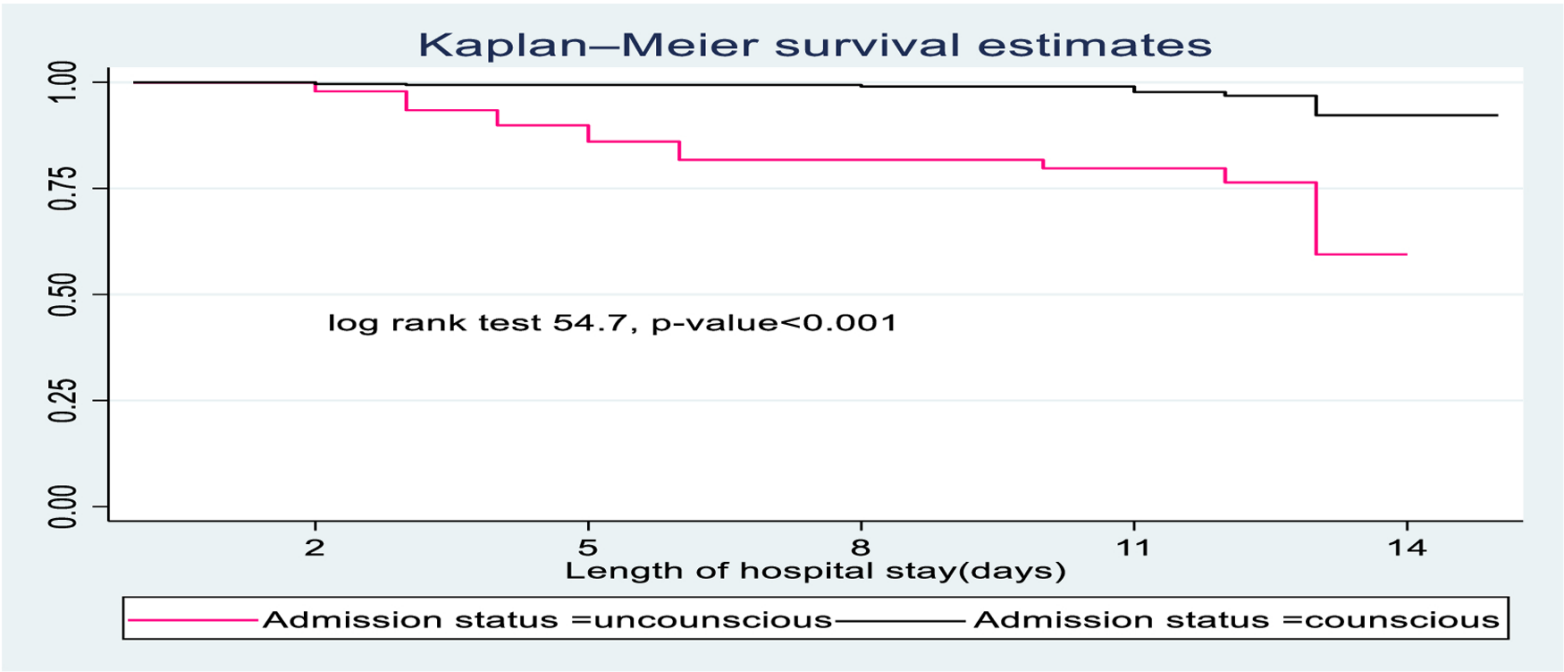
The Kaplan-Meier survival curves compare the survival time of SCAP children admitted with categories of status at admission to pediatrics ward of DTCSH, from 2021 to 2022, North central Ethiopia, 2022.

In this study, SCAP children with impaired consciousness levels during admission had lower survival time compared to conscious children. On the 15th day of the hospital stay, the cumulative survival probability of SCAP children who had altered consciousness was 51.4% compared to their counterparts with 89.4% (Figure 2).

**Figure 3:**
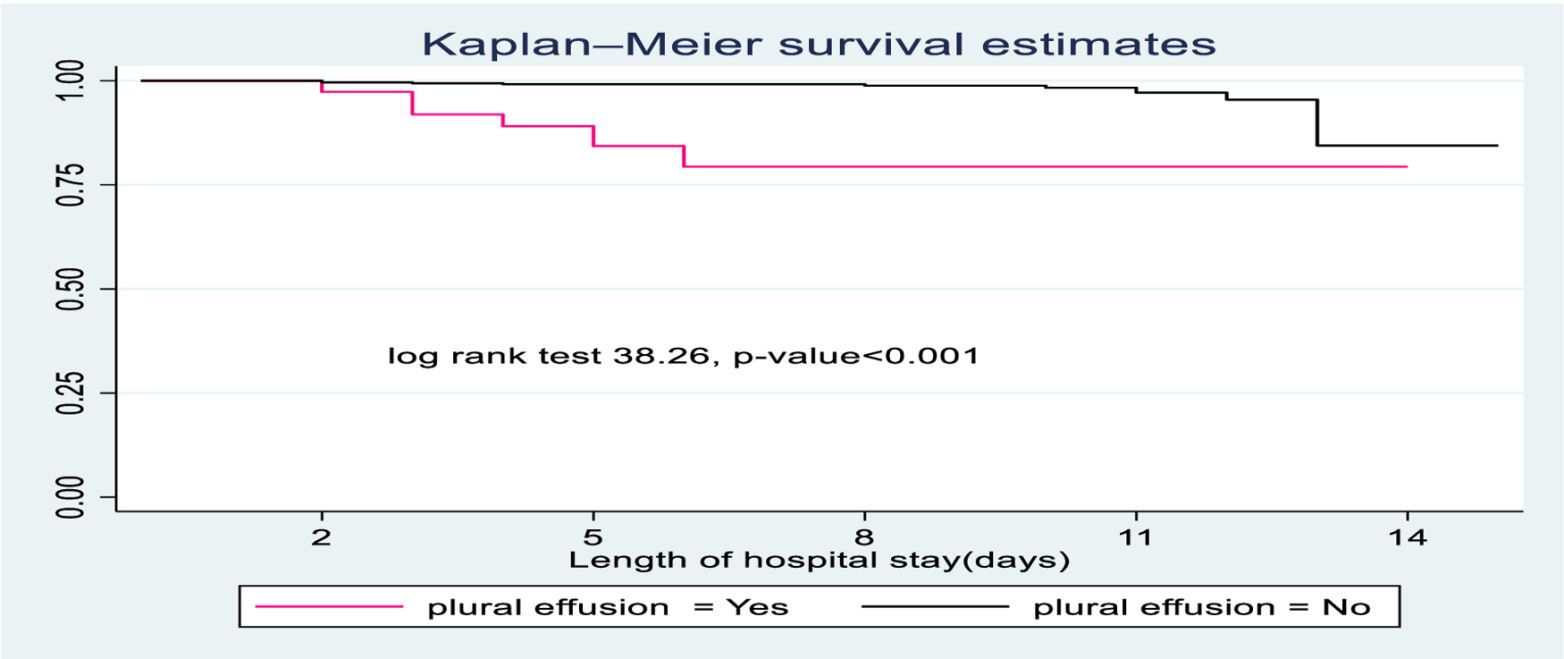
The Kaplan-Meier survival curves compare the survival time of SCAP children admitted with categories of pleural effusion to pediatrics ward of DTCSH, from 2021 to 2022, North central Ethiopia, 2022.

SCAP children diagnosed with pleural effusion had less survival probability than those who had no pleural effusion. The overall survival at the end of the follow-up period was 77.3% for those who had pleural effusion compared to children with no pleural effusion (89.35) (Figure 3).

**Figure 4:**
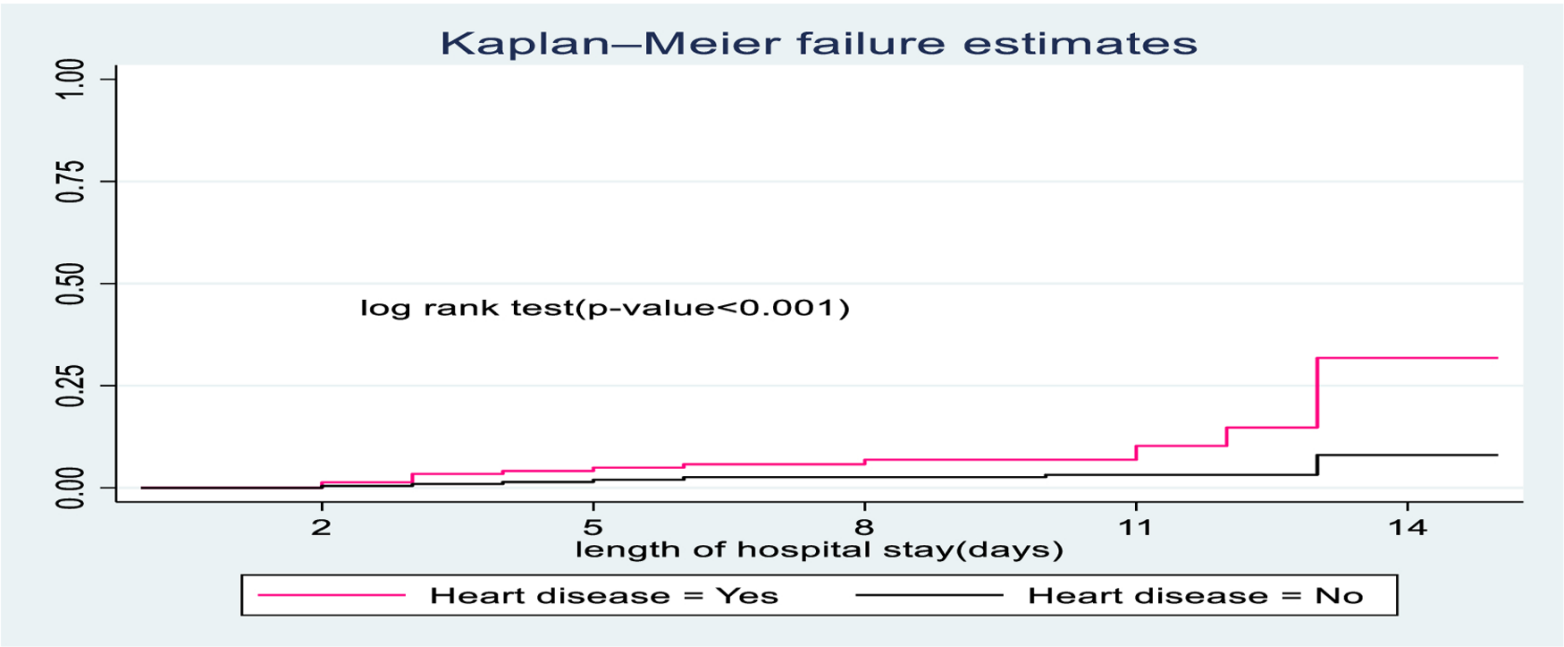
The Kaplan-Meier hazard curves compare hazard time of SCAP children admitted with categories of heart disease to pediatrics ward of DTCSH, from 2021 to 2022, North West Ethiopia, 2022.

The survival time of children with no heart disease was longer than children suffering from heart disease. At the end of the study, the probable hazard of death for those children with heart disease was 39.7% compared to their counterparts with 10.5% (Figure 4).

#### Survival status of children diagnosed with severe community acquired pneumonia

A total of 580 study participants were followed for different periods: a minimum of 2 and a maximum of 15 days, and the overall median hospital stay was 8.2 days; of all 580 study subjects, 27 (4.7%) were died (CI: 3.1, 6.7) and 553 (95.3%) censored (among them 451 (77.8 % recovered), 55 (9.1% referred), 28 (4.8% defaulted) and 3.6% were against medical advice at the end of the study period).

#### Predictors of mortality among children with severe community acquired pneumonia

In bi-variable Cox proportional hazard regression analysis, 17 variables (sex, educational level of mothers, number of family size, heart disease, pleural effusion, anemia, first time wheeze, Pertusis, malaria, chest indrawing, temperature at admission, pulse rate at admission, treatment failure, previous admission of SCAP, history of developmental delay, WFL and status at admission) were significantly associated with time to death. However, after a running multivariable Cox proportional hazard regression analysis, only five variables (heart disease, previous admission of SCAP, WFL < −3Z score, impaired consciousness at admission and pleural effusion) were found to be significant predictors of mortality.

The result of multivariable analysis showed that Children with pleural effusion showed a hazard of death of 3.42 compared to their counterparts (AHR: 3.42; 95%CI: 1.18–9.93). Likewise, the risk of mortality of children who were previously admitted for SCAP was 3.87 times that of their counterparts (AHR: 3.87; 95% CI: 1.31, 11.43) (Table 5).

**Table 5:** Bi-variable and multivariable Cox regression analysis results showing the association of variables with time to death among children with SCAP admitted at DTCSH, North central Ethiopia, 2022 (*n* = 580).

Variables		Treatment outcome		CHR(95% CI)	AHR(95%C I)
		Event (%) censored (%)			
Sex of the child	Male	16 (5.5)	276 (94.5)	1.50 (.70–3.23)	1.40 (.57–3.46)
	Female	11 (3.8)	277 (96.2)	1	1
Treatment failure	Yes	20 (11.8)	149 (88.2)	6.70 (2.82–15.90)	1.24 (.370–4.16)
	No	7 (1.7)	404 (98.3)	1	1
Anemia	Yes	13 (13.0)	87 (87.0)	3.95 (1.85–8.44)	1.37 (.43–4.33)
	No	14 (2.9)	466 (97.1)	1	1
WFL	<−3 Zscore	23 (7.8)	271 (92.2)	5.65 (1.95–16.37)	3.57 (1.02–12.42)*
	≥−3 Zscore	4 (1.4)	282 (98.6)	1	1
Chest in drawing	Yes	20 (8.9)	204 (91.1)	4.33 (1.83–10.26)	1.55 (.52–4.60)
	No	7 (2.0)	349 (98.0)	1	1
Pleural effusion	Yes	14 (18.7)	61 (81.3)	7.32 (3.44–15.59)	3.42 (1.18–9.93)*
	No	13 (2.6)	492 (97.4)	1	1
Pertussis	Yes	7 (7.3)	89 (92.7)	2.08 (.88–4.95)	0.69 (.225–2.14)
	No	20 (4.1)	464 (95.9)	1	1
Previous Admission of SCAP	Yes	21 (14.5)	124 (85.5)	9.52 (3.83–23.67)	3.87 (1.31–11.43)*
	No	6 (1.4)	429 (98.6)	1	1
First time wheeze	Yes	13 (8.2)	145 (91.8)	2.30 (1.08–4.91)	1.52 (.63–5.29)
	No	14 (3.3)	208 (96.7)	1	1
Heart disease	Yes	15 (10.0)	135 (90.0)	3.37 (1.57–7.21)	4.37 (1.68–11.32)*
	No	12 (2.8)	418 (97.2)	1	1
Educational level of mothers	Unable to read and write	12 (7.6)	145 (92.4)	1.13 (.32–4.04)	0.60 (.14–2.63)
	Primary(1–4)	4 (3.8)	101 (98.2)	0.61 (.14–2.72)	0.44 (.08–2.43)
	Primary(5–8)	3 (1.8)	166 (98.2)	0.33 (.07–1.64)	0.55 (.09–3.19)
	Secondary	5 (95.0)	94 (5.0)	0.77 (.18–3.27)	0.41 (.08–2.18)
	College &above	3 (6.0)	47 (94.0)	1	
Pulse rate at admission	Altered	19 (5.6)	319 (94.4)	1.55 (.68–3.56)	1.84 (.71–4.77)
	Normal	8 (3.3)	234 (96.7)	1	1
Temperature at admission	Altered	22 (7.1)	289 (92.3)	3.54 (1.34–9.37)	2.15 (.68–6.75)
	Normal	5 (1.9)	264 (98.1)	1	1
Status at admission	Altered	19 (20.2)	75 (79.8)	11.46 (4.98–26.33	3.41 (1.14–10.19)*
	Conscious	8 (1.7)	478 (98.4)	1	1
Malaria	Yes	8 (8.5)	86 (91.5)	1.93 (.84–4.43)	0.81 (.29–2.28)
	No	19 (3.9)	467 (96.1)	1	1
Number of family size	<3	5 (4.4)	109 (95.6)	0.89 (.30–2.61)	2.87 (.745–11.04)
	3–5	12 (5.2)	220 (94.8)	1.21 (.52–2.79)	3.01 (.77–12.22)
	>5	10 (4.3)	224 (95.7)	1	1
Developmental delay	Yes	3 (3.0)	96 (97.0)	0.51 (.15–1.71)	0.39 (.10–1.47)
	No	24 (5.0)	457 (95.0)	1	1

* NB. significant (*p*-value less ≤0.05); CHR, crude hazard ratio; AHR, adjusted hazard ratio; CI, confidence interval; 1, as reference categories.

## Discussion

The current study aimed to determine the survival status and predictors among under-five children diagnosed with SCAP. Heart disease, previous admission of SCAP, WFL < −3Z score, impaired consciousness at admission, and pleural effusion were significant predictors. The overall incidence density rate was 5.7/1,000 child-days.

This study disclosed that the overall mortality of children diagnosed with severe community-acquired pneumonia was 4.7% (95% CI: 3.10, 6.70) during the follow-up period. The finding of this study was higher than a study done in Debre Markos referral hospital (19).The possible justification could be attributable to differences in socio-economic status, sample sizes, and also variation in presence of increased co-morbid cases as it increases mortality rate. On the other hand, this finding is lower than a study done in University of Gondar Comprehensive Specialized Hospital (3). This difference could be due to the methodology, strictly adhering to updated guideline for pneumonia management and staff training which increased quality of care provided for SCAP children (4), health seeking behaviors and delayed presentation to healthcare facilities, study settings, and time variation in which the studies were conducted and availability of medications might also the cause of variation.

This study revealed that SCAP children with severe wasting (WFL < −3Z score) were 3.57 times more vulnerable to death compared to children with no severe wasting (WFL ≥ −3Zscore). This result is supported by a study done in Khammam (22), India (23). The possible justification could be that malnutrition increases the frequency and severity of pneumonia, delays recovery, and increases risk of death (24). Acute infections like pneumonia in return worsen nutritional status through higher catabolism, loss of appetite, and nutrient loss, contributing to a worsening cycle of infections and malnutrition. Moreover, malnutrition is not only associated with an increased risk of pneumonia episodes but also increased severity and case fatality (25).

SCAP children who had heart diseases were at a higher danger of death compared to their counterparts. This finding is in line with a study conducted in Chennai (21). The justification could be pneumonia patients co-morbid with heart diseases (congestive heart failure and congenital heart disease) had an increased risk of further infection and prolonged ventilatory support associated with high rates of complications, including sepsis, shock, and death (26). Pneumonia could also precipitate the presence and progression of heart failure. The pathophysiology might be attributable to fluid-filled alveoli in congestive heart failure that reduced oxygenation and organism clearance by interfering with macrophage function (27).

This study also investigated children with pleural effusion and found they had a 3.42 times likelihood of death compared to children with no pleural effusion. A possible reason could be infectious pleural effusion, which is caused by pneumonia and is the most common cause leading to serious complications such as empyema or other serious complications leading to high rates of mortality and low survival (28).

The risk of mortality in children who were previously admitted for SCAP showed a 3.87 times higher danger of death compared to their counterparts. This is justified by children who have been hospitalized previously due to similar diagnoses (SCAP), as they are more prone to further complications, resistance of medications (29), and psychological disturbance of both child and parents, which in turn further compromises the immune system; readmitted patients are exposed for further hospital acquired infections and life-threatening complications. Readmission is also challenging for families in terms of healthcare costs, as it can be unaffordable to buy expensive drugs. All these increase the severity and case fatality rate of children with SCAP (30).

Furthermore, the current study showed that children whose consciousness levels altered at admission were 3.41 times more likely to die compared to conscious chidden. This result is in line with a study done in Chennai (21). This might be because of unconscious children unable to feed and drink that are exposed to alternative feeding methods, intravenous medication, and fluids which increase the management complexity, further reducing chances of survival and increasing mortality (31). Although this study used a prospective cohort study design which included socio-demographic and laboratory related factors and enables the relationship between the exposure and outcome variables it has some limitations. Some variables were not accessible in the medical records, and children admitted without mothers/caregivers were excluded from the study, which may affect the outcome variable and underestimate the result.

## Conclusion

In this study, the survival probability was low with an overall mortality rate of 4.7/1,000 child-days. Children with heart disease, previous admission of SCAP, WFL < −3Z score, an impaired consciousness level at admission, and pleural effusion displayed low survival. Therefore, placing special emphasis on and providing close follow-up for children with SCAP—particularly those with identified predictors—will increase survival. In addition, a multi-center study is recommended.

## Data Availability

The original contributions presented in the study are included in the article/**Supplementary Material**, further inquiries can be directed to the corresponding author/s.
