# Supplementary material for: Survival status and predictors of mortality from severe community-acquired pneumonia among under-five children admitted at Debre Tabor comprehensive specialized hospital: a prospective cohort study
Source: Front Pediatr. 2023 Jun 5;11:1141366. doi: 10.3389/fped.2023.1141366 (PMC10280987; doi:10.3389/fped.2023.1141366)
Supplement: Supplementary file 1 [file Datasheet1.pdf]

Operational definitions

Admission respiratory rate: admission respiratory rate of the child as **altered** when it was tachypnea or bradypnea based on age. That means, when the child admitted with low respiratory rate or bradypnea (below the normal range based on age) and the child admitted with high respiratory rate or tachypnea (above the normal range based on age) was decided as **altered**, otherwise normal

Admission pulse rate: admission pulse rate of the child as **altered** when it was tachycardia or Bradycardia based on age. That means, when the child admitted with low pulse rate or Bradycardia (below the normal range based on age) and the child admitted with high respiratory rate or tachycardia (above the normal range based on age) was decided as **altered,** otherwise normal

Admission temperature: admission temperature of the child was classified as **altered** when it was above or below from the normal range, otherwise normal

Fast breathing: It is classified based on age. RR>60 for children birth up to 2 month, RR>50 2onth up to 12 month, RR>40 12 month up to 5years as Fast breathing, other wise normal

Up to date immunizations: children currently (during data collection) on immunizations based on the schedule.

Fully vaccinated: : Full vaccination includes all children who had obtained BCG (bacillus calmette–gue´rinvaccine) and OPV0 (oral polio vaccine) at birth, pentavalent1 (DPT-hepB, Hib [diphtheria, Pertussis, tetanus, hepatitis B and Haemophilus influenza type b]), OPV1, Rota1 and PCV1, at 6weeks; pentavalent 2, OPV2, Rota2, PCV2 at 10weeks; pentavalent 3, PCV3, IPV at 14 weeks; and measles vaccine at 9 and 15 months[11]

TB: children admitted with SCAP and co-infected with PTB. It might be Child on a Treatment, or positive for screening during admission defined as SCAP co-morbid with TB

Cardiac disease: children with SCAP and co-morbid with Cardiac disease (CHF, RHD, congenital heart disease basically, VSD, ASD or TOF)

Vitamin use: SCAP children were asked the history of taking vitamins as a vaccination (Vit A) or as treatment Vit D and vit A

Altered consciousness: children with SCAP admitted with impaired consciousness (lethargy, coma)

Danger signs: loss of consciousness, abnormal body movement, vomiting everything, convulsion, inability to feed in addition to SCAP

Co-morbidity: any disease condition (acute or chronic) present at admission in addition to SCAP which includes hyperactive airway disease (childhood asthma), retroviral infection, Tuberculosis, acute gastroenteritis, Pertussis, anemia, meningitis, measles, bronchitis, heart disease, and urinary tract infection
